# Supplementary material for: A nanostructured surface increases friction exponentially at the solid-gas interface
Source: Sci Rep. 2016 Sep 6;6:32996. doi: 10.1038/srep32996 (PMC5011718; doi:10.1038/srep32996)
Supplement: Supplementary Information [file srep32996-s1.pdf]

Supplementary Materials

A nanostructured surface increases friction exponentially at the solid-gas interface

Arindam Phani[1*], Vakhtang Putkaradze[2*], John E. Hawk[1], Kovur Prashanthi[1], Thomas Thundat[1*]

[1]*Department of Chemical and Materials Engineering,* [2]*Department of Mathematical Statistical Sciences, University of Alberta, Edmonton, Alberta T6G 1H9, Canada*

*Correspondence to *phani@ualberta.ca*, *putkarad@ualberta.ca*, *thundat@ualberta.ca*

S1. FE-SEM of nanostructured surface

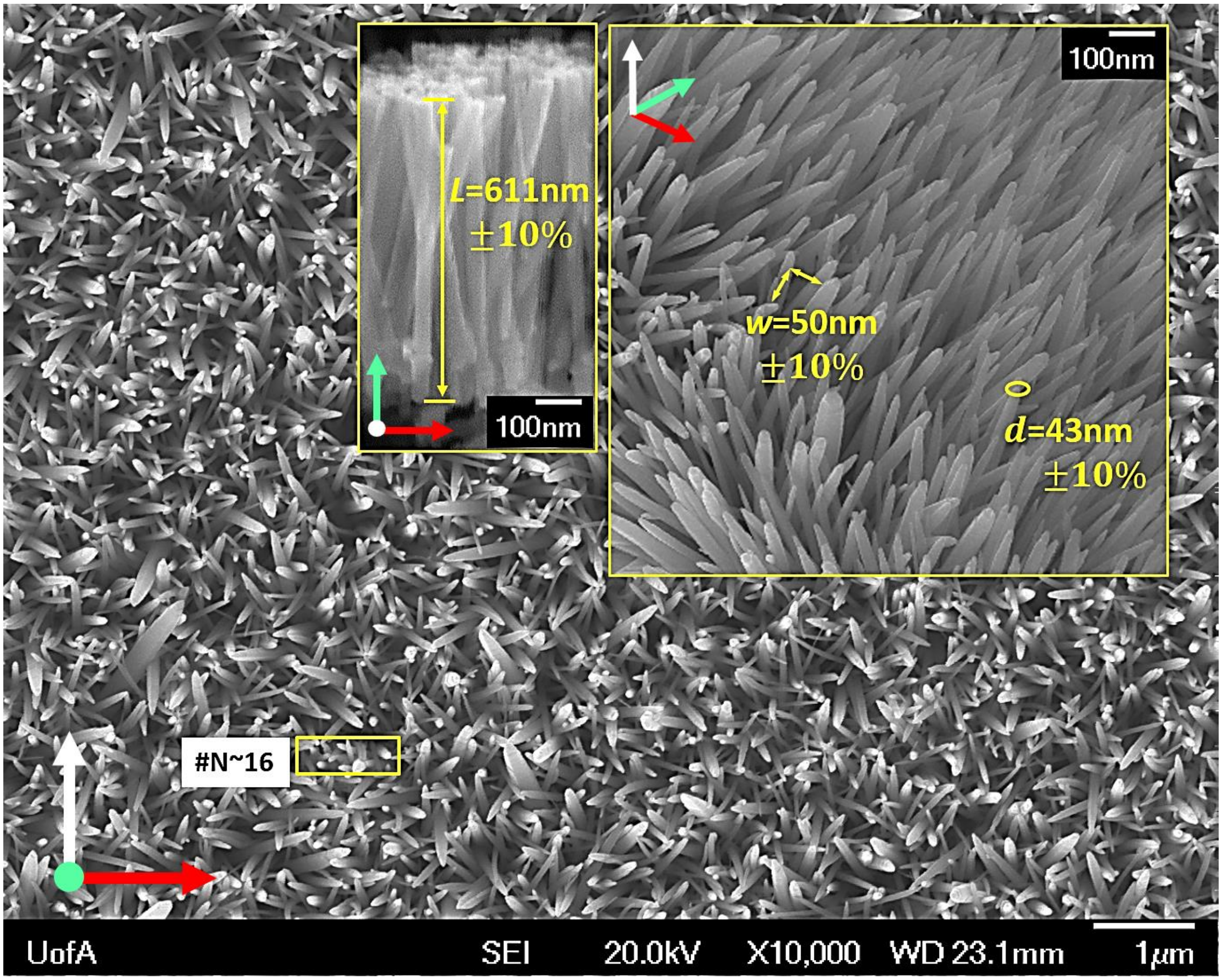

Figure S1| FE-SEM image of nanostructured QC with embedded scale-bars.

S2. XRD analysis of nanostructured surface:

Fig. S2 presents the XRD analysis of ZnO nanorods grown on QC. All the diffraction peaks could be indexed to hexagonal phase of ZnO with polycrystalline grain orientations (JCPDS: 36-1451). No characteristic peaks from other impurities are detected, which indicates the purity of grown ZnO nanorods. The Au (200) peak corresponds to Au layer on QC. Since the ZnO nanorods are polycrystalline in nature, the piezoelectricity of ZnO nanorods is insignificant due to mutual cancellation of induced strain, therefore, less likely to impact the dynamic response of the QC resonator.

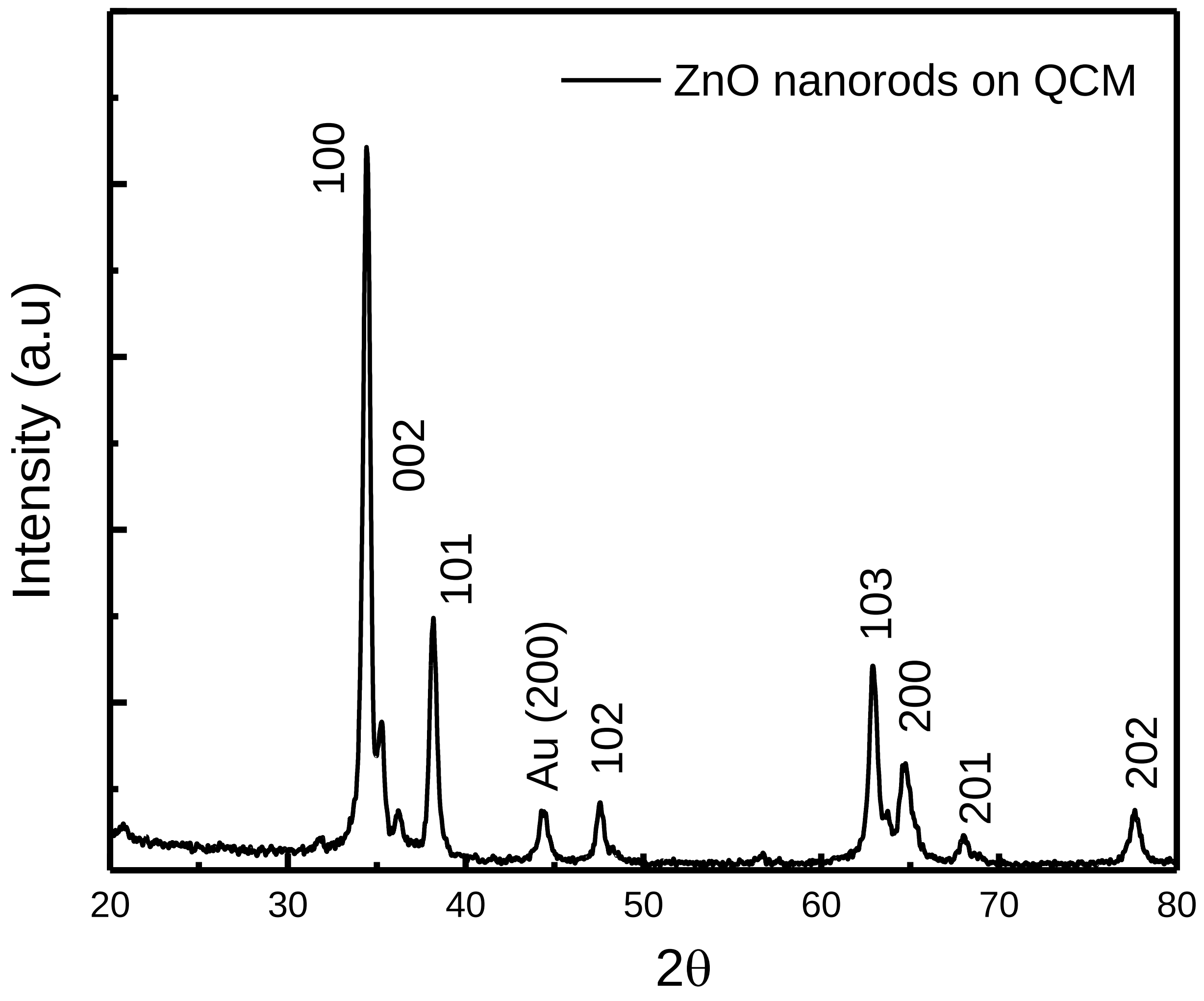

Figure S2| XRD-analysis of nanostructured QC.

S3. Measured data accuracy analysis and estimation

Conventional models[1–4] regard the QC as an electrical resonator obeying the equation of a driven damped simple harmonic mechanical oscillator

$$\ddot{x} + \gamma\dot{x} + \omega_0^2 x = A\cos(\omega t) \qquad \text{(S1)}$$

where we have denoted resonant frequency $\omega_0 = \sqrt{k/m_{eff}}$, k the stiffness, γ the effective dissipation coefficient and m_{eff} the effective mass with A and ω, the drive amplitude and frequency respectively. In the electrical analogue, x acquires the meaning of electric charge q, γ the relative resistance $\wp$ per unit inductance ℓ and spring stiffness defined as $k = 1/c$ with c being the capacitance.

The simultaneous measurement of damping factor γ and resonance frequency f_{QC} allows the estimation of non-dimensional damping D as $D = \frac{\gamma_{\max}}{f_{QC}}$. We shall note here that the dissipation D is measured directly from the experiments as illustrated in Fig. 1a in main text, whereas Q-factor is estimated from the width of the Lorentzian peak of the amplitude response (Fig. S3 below). From general theory, one expects that, independent of the type of measurement, Q=1/D. This is substantiated by the plot of normalized values of Q vs $1/D$, with data points lying on a straight line with slope 1 (Fig S4). The highest intercept value, and deviation of the slope from 1, obtained in the fits corresponds to the accuracy in the experiments ~10^{-7}. This attests to the high accuracy of our experiments.

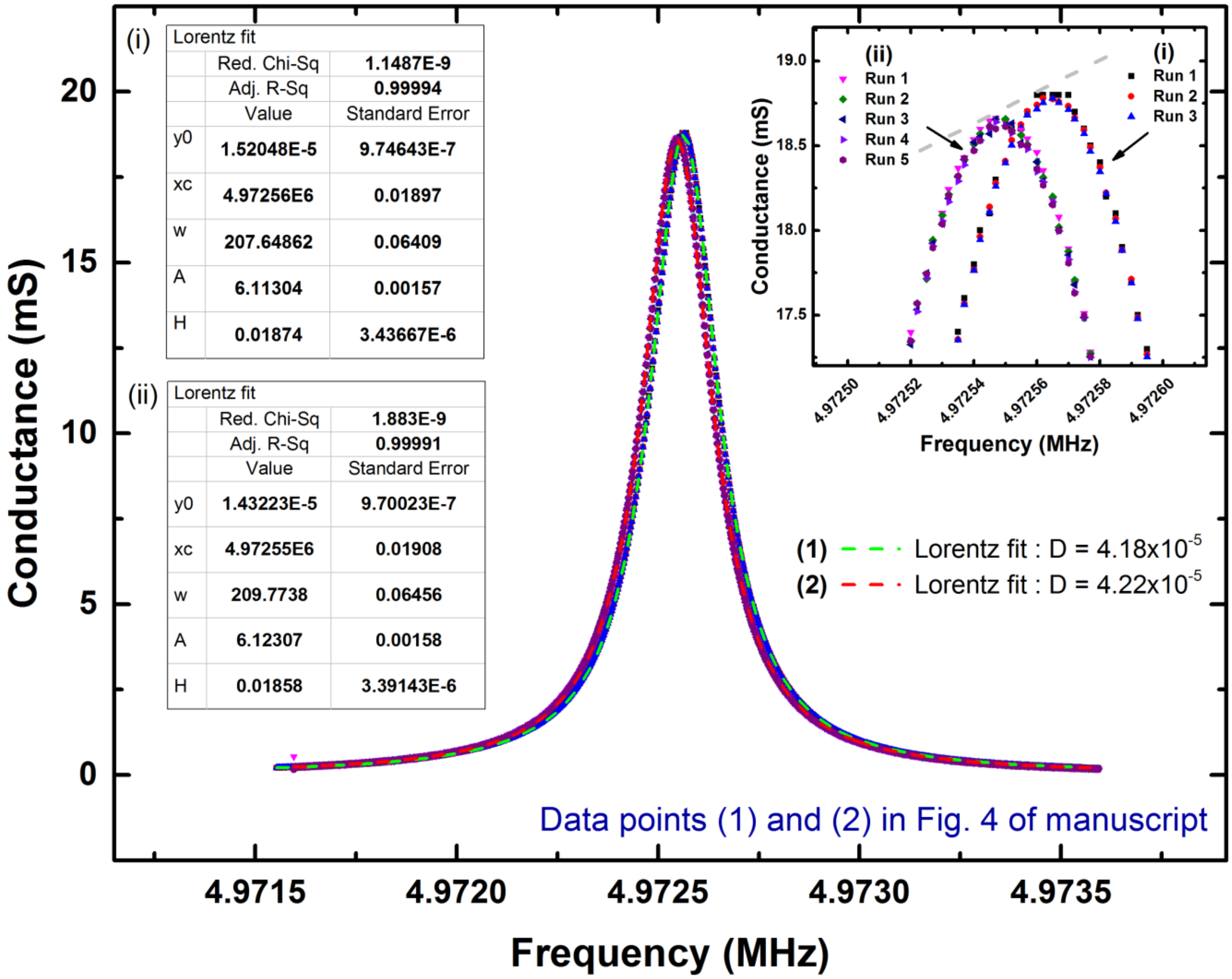

Figure S3| Conductance vs Frequency response of the Nanostructured QC resonator for small changes in input drive energy.

Figure S3 shows the Lorentzian fit for a particular measurement giving an estimate of the Q-factor. Data points for the Q factor and dissipation D from experiments have been analyzed in this way to estimate measurement accuracy, as represented in Fig. S3 above and for generating Fig. 2a in the main article.

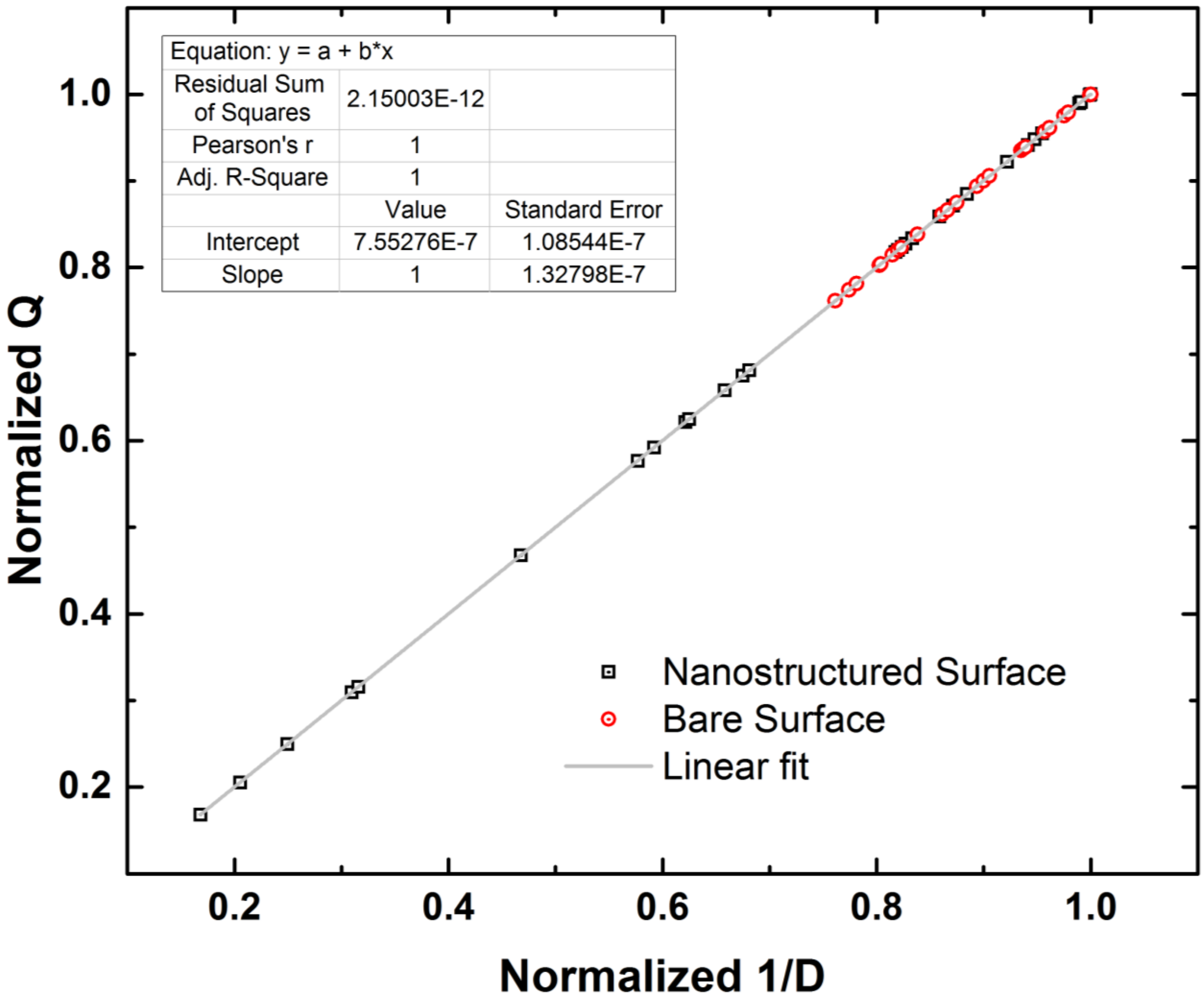

Figure S4| Accuracy analysis of measured data

S4. Dissipation responses for different gas media

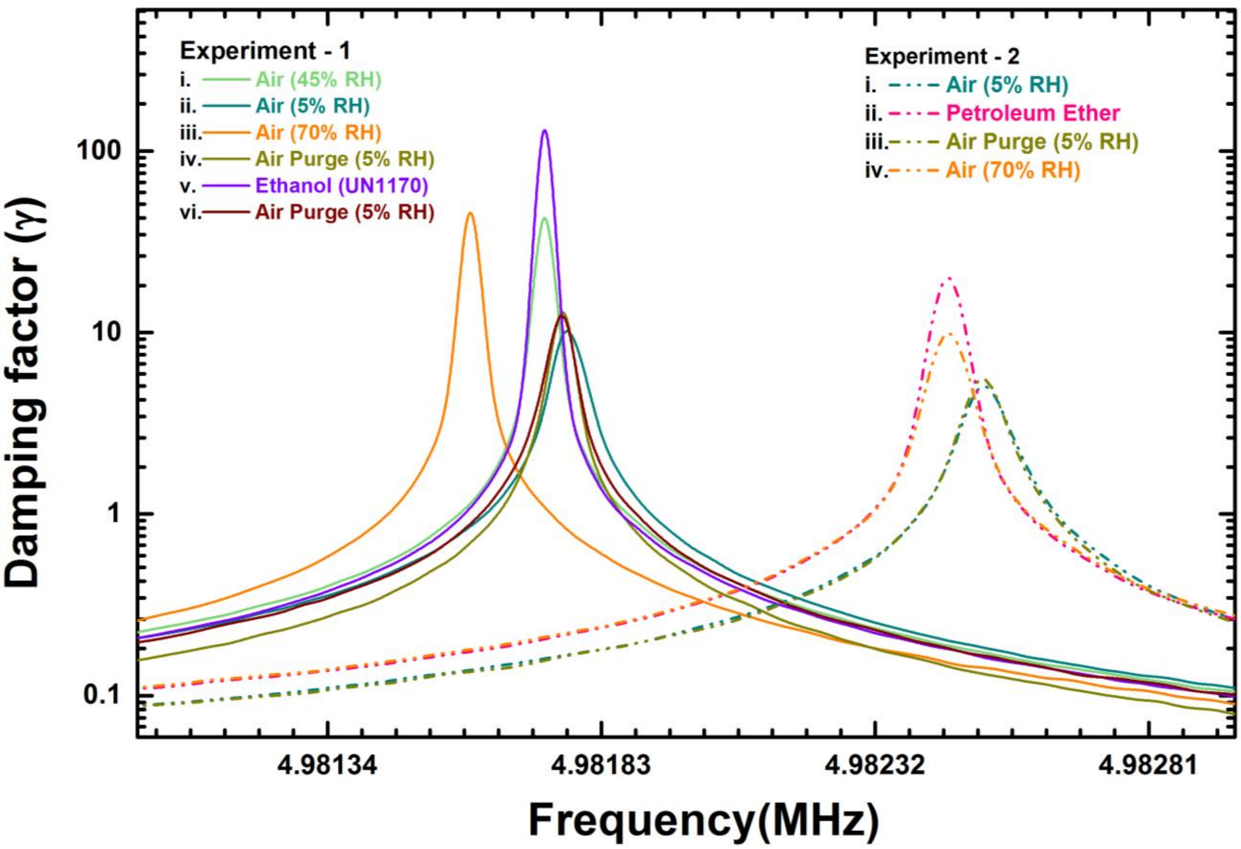

Figure S5| A representative set of dissipation measurements for different gas media as done in sequence. Note that each graph is obtained as a concatenated fit of multiple readings similar to that presented in Fig. S3 above.

S5. Estimation of gas mixture viscosities

There are two possible ways to bring about a change in the viscosity ν as a function of the media properties. One way is to change the medium, for example by saturating the air with the vapor of a chemical up to a particular concentration level (ppm levels in our case), or, alternatively, replacing the air altogether by another gas. In the case of complete replacement, the effective variation in ν of the medium is relatively easier to compute following

$$\frac{v_{vapour}}{v_{dry-air}} = \left(\frac{\rho_{dry-air}}{\rho_{vapour}}\right) \cdot \left(\frac{\eta_{vapour}}{\eta_{dry-air}}\right) \tag{S2}$$

For mixtures however, it is more complex. The changes in viscosity v_m in a mixture can be estimated from well-established theoretical and experimental work on the subject[5–9]. For a complex vapor mixture of air and volatile chemicals, air molecules of the fluid medium around the nanorods are replaced by molecules of the vapor, depending upon its partial vapor pressure and hence its number density and molar mass, which results in an effective change in kinematic viscosity Δv. The experimental viscosity mixture of up to 3 components v_m may be calculated using[6]

$$v_m = \frac{v_1}{1 + \frac{1-x_1}{x_1}\left(\frac{1.385 v_1}{\rho_1 D_{1m}}\right)} + \frac{v_2}{1 + \frac{1-x_2}{x_2}\left(\frac{1.385 v_2}{\rho_2 D_{2m}}\right)} + \frac{v_3}{1 + \frac{1-x_3}{x_3}\left(\frac{1.385 v_3}{\rho_3 D_{3m}}\right)}\ , \tag{S3}$$

with x_i being the mole fraction of component i determinable from the vapor pressure at normal temperature and pressure conditions, ρ_i being the vapor density of component i. The diffusion coefficient D_{im} of component i into a mixture of *2 and 3* is defined as

$$D_{im} = \frac{1-x_i}{\frac{x_2}{D_{i2}} + \frac{x_3}{D_{i3}}}\ , \tag{S4}$$

as derived from Maxwell's equations for diffusion[6]. The diffusion coefficient of two component mixture is derivable from[10]

$$D_{12} = \frac{1.858 \times 10^{-3} T^{3/2} \sqrt{\frac{1}{M_1} + \frac{1}{M_2}}}{p \sigma_{12}^2 \Omega_c} \tag{S5}$$

where, p is the pressure in atm. , M is the molar mass, T is the absolute temperature in Kelvin and $\sigma_{12} = 1/2\left(\sigma_1 + \sigma_2\right)$ is the average collision diameter. Also, Ω_c is the temperature dependent collision integral and is assumed to be 1 in our calculations considering non-interacting molecules. The values of σ_i are obtained from literature data[8] or computed based on molar volume and density.

S6. Estimation of gas viscosity at different temperatures

The variation of kinematic viscosity as a function of temperature is expected and has been a subject of interest for many years. The kinematic viscosity of air for different temperatures as in our experiments are computed from[11] using 120 as the Sutherland's constant.

S7. Estimation of vibration amplitude at 5mV drive from impedance measurement

The key to understanding our results and theoretical formulation lies in the fact that in the parameter regime we are operating, which is indeed described in Reference[3], the amplitude is proportional to the measured conductance G ($\mathrm{Re}(Y)$, the *Admittance*). More precisely, from equation (2) in Reference[3], page 4519 and using our notation, (see also Appendix section in Reference[4]) we conclude that $a \propto G$. Thus, the quantity $\Delta a/a$ is *exactly equal* to $\Delta G/G$. In our experiment (using their notation), G is measured with a ppm accuracy by the impedance analyzer as

$$G = \frac{2\phi}{V}\dot{u} = \frac{2\phi}{V} fu, \qquad \text{(S6)}$$

where V is the drive voltage across the crystal, f is the frequency at resonance, u is the lateral displacement (a in our case) at the crystal surface, $\dot{u}$ is the lateral speed, and $\phi = \frac{A_q e_{26}}{d_q}$ is a crystal factor. The consideration of conductance G , the $\mathrm{Re}(Y)$ is valid here since the entire analysis is done with respect to magnitudes at zero phase at resonance.

The crystal shear amplitude or lateral surface amplitude a in our representations of resonance amplitude can be estimated from impedance measurements as has been discussed in literature[3,4] using appropriate parameters. A quick calculation using values for our used crystal: $A_q = 4.084\times10^{-6} m^2$ (equivalent electrode surface area, with 12% per μm^2 (Fig. S1) coverage with nanorods), $e_{26} = 9.54\times10^{-2} Cm^{-2}$ [3] and $d_q = 333\times10^{-6} m$,

$$2\phi = 2\times\frac{4.084\times10^{-6} m^2 \times 9.54\times10^{-2} Cm^{-2}}{333\times10^{-6} m} = 0.002337\, Cm^{-1}. \qquad \text{(S7)}$$

From measured conductance $G \sim 18.5\times10^{-3}$ S (Fig. S3) at 5mV input drive, and at resonance $f = 4.972 MHz$,

$$u = a = \frac{1}{2} \times \frac{18.5 \times 5 \times 10^{-6}}{0.002337 \times 4.972 \times 10^{6}} = \frac{1}{2} \times 7.521 \times 10^{-9} m = 3.9 nm \sim 4 nm . \quad \text{(S8)}$$

The final factor of $1/2$ is for considerations of the motion of the nanorod with respect to its center axis as relevant to our oscillating nanorod theoretical model. This is the estimate used in our theoretical model, providing the excellent match to the experimental data without the need of fitting parameters.

S8. Explanation of exponential amplitude dependence

Equation (2) in text can also explain the exponential nature of D with respect to the changes in the amplitude at resonance. If we assume the simplest possible linear dependence $a_{eff} = w + z\Delta a$, with Δa being deviation from the smallest resonance amplitude for the lowest input drive energy, equation (2) yields, for small changes of Δa

$$D = D_0 \exp\left[2K \left(1 - \frac{a}{w} z \right) \frac{\Delta a}{a} \right] = D_0 \exp\left[-\lambda \frac{\Delta a}{a} \right], \quad \text{(S10)}$$

where $\lambda = 2K\left(\frac{a}{w} z - 1 \right)$, z being a non-dimensional fitting parameter in experiments and D_0 being the dissipation for the smallest amplitude. Further development of the theory can be done, assuming more accurate expression for a_{eff} that is nonlinear in $\left(\frac{\Delta a}{a} \right)$, giving essentially the same results.

REFERENCES

1. Rodahl, M. *et al.* Simultaneous frequency and dissipation factor QCM measurements of biomolecular adsorption and cell adhesion. *Faraday Discuss.* **107,** 229–246 (1997).

2. Rodahl, M., Höök, F., Krozer, A., Brzezinski, P. & Kasemo, B. Quartz crystal microbalance setup for frequency and Q-factor measurements in gaseous and liquid environments. *Rev. Sci. Instrum.* **66,** 3924 (1995).

3. Johannsmann, D. Viscoelastic, mechanical, and dielectric measurements on complex samples with the quartz crystal microbalance. *Phys. Chem. Chem. Phys.* **10,** 4516–4534 (2008).

4. Kanazawa, K. K. Mechanical behaviour of films on the quartz microbalance. *Faraday Discuss.* **107,** 77–90 (1997).

5. Brokaw, R. S. *NASA Technical Note: Viscosity of gas Mixtures*. (1968).

6. Buddenberg, J. W. & Wilke, C. R. Calculation of Gas Mixture Viscosities. *Ind. Eng. Chem.* **41,** 1345–1347 (1949).

7. Davidson, T. A. *A Simple and Accurate Method for Calculatlng Viscosity of Gaseous Mixtures*. (1993).

8. Neufeld, P. D. Empirical Equations to Calculate gas mixture viscosities as a function of the Transport Collision Integrals Ω. *J. Chem. Phys.* **57,** 1100 (1972).

9. Reid, R. C., Poling, B. E. & Prausnitz, J. M. *The Properties of Gases and Liquids*. (McGraw-Hill, 1987).

10. Cussler, E. L. *Diffusion mass transfer in fluid systems*. (Cambridge University Press, 1997).

11. Sutherland, W. LII. The viscosity of gases and molecular force. *Philos. Mag. Ser. 5* **36,** 507–531 (1893).
